# Factors associated with problematic drug use among psychiatric outpatients**^1^**

**DOI:** 10.1590/1518-8345.1444.2815

**Published:** 2016-11-28

**Authors:** Clarissa Mendonça Corradi-Webster, Edilaine Cristina da Silva Gherardi-Donato

**Affiliations:** 2 PhD, Professor, Faculdade de Filosofia, Ciências e Letras de Ribeirão Preto, Universidade de São Paulo, Ribeirão Preto, SP, Brazil.; 3 PhD, Associate Professor, Escola de Enfermagem de Ribeirão Preto, Universidade de São Paulo, PAHO/WHO Collaborating Centre for Nursing Research Development, Ribeirão Preto, SP, Brazil.

**Keywords:** Mental Disorders, Substance-Related Disorders, Comorbidity

## Abstract

**Objective:**

to examine the factors associated with problematic drug use among psychiatric outpatients.

**Method:**

a cross-sectional study was carried out in two mental health services. Eligible individuals were patients of these mental health services, who used them within the data collection period. Instruments: standardized questionnaire with sociodemographic, social network, social harm, and clinical information; Alcohol, Smoking and Substance Involvement Screening Test; Barratt Impulsiveness Scale; Holmes and Rahe Stress Scale. Statistical analysis was performed using parametric statistics considering a significance level of p ≤ 0.05. Study participants were 243 patients, with 53.9% of these presenting problematic drug use.

**Results:**

the most important independent predictors of problematic drug use were marital status (OR = 0.491), religious practice (OR = 0.449), satisfaction with financial situation (OR = 0.469), having suffered discrimination (OR = 3.821) and practicing sports activities in previous 12 months (OR = 2.25).

**Conclusion:**

the variables found to be predictors were those related to the social context of the patient, there, it is recommended that mental health services valorize psychosocial actions, seeking to know the social support network of patients, their modes of socialization, their financial needs, and their experiences of life and suffering.

## Introduction

A growing body of research has shown that the prevalence of problematic drug use is high among people with psychiatric disorders^1^. In the previous decades, there has been an increasing interest in the study of the prevalence and characteristics of dual diagnosis, as it has been argued that dual diagnosis patients present higher morbidity, poorer prognoses and more clinical treatment difficulties^2^ ^-^ ^3^. The literature indicates that some sociodemographic characteristics may be associated with this dual pathology. These mainly include gender^3^ ^-^ ^4^, marital status^5^, age^5^, educational level^3^ ^,^ ^5^, employment^5^, and social support^3^. It also indicates that clinical characteristics may be associated, these being treatment adherence^3^ ^-^ ^4^, number of hospital admissions^3^ ^-^ ^4^, and family history of substance use^5^. It is important to note that these studies were conducted in other countries, with no references found for studies in Brazil that were designed to identify variables associated with drug use by individuals undergoing treatment in community mental health services. 

In Brazil, as in the majority of countries, the psychosocial care network is divided between services that specialize in problems related to drug use and mental health services^6^. Thus, people with a dual diagnosis, in which the drug problem is evident, are quickly referred to services that specialize in treatment for drug users. However, there are a considerable number of drug users in mental health services whose consumption is not the main complaint, with this representing a problem coping strategy or even being a way of dealing with the adverse effects of the medication therapy. These people are usually not identified as problematic users or, when identified, their consumption is not considered by professionals to be justification for referral. Despite the dual diagnosis leading to a worse prognosis, the literature indicates that mental health services tend not to investigate this association, failing to act preventively and offer comprehensive treatment^7^. Greater investment is suggested in the training of mental health professionals to instrumentalize them in caring for this population, and efforts are recommended to improve the pharmacological and nonpharmacological therapeutic approaches^6^. 

With the aim of creating guidelines for these services, it is necessary to have a panorama of the problem of drug use that reflects the reality of a diverse clientele existing in community mental health services in Brazil. For this, further knowledge about the social setting, socio-demographic and clinical characteristics of psychiatric outpatients who problematically use drugs is important to guide intervention studies and therapeutic actions in mental health services. This study aimed to examine the factors associated with problematic drug use among psychiatric outpatients of community mental health services.

## Methods

The study had a cross-sectional design, and was carried out in two mental health services of Ribeirão Preto, the eighth largest city of the state of São Paulo, Brazil, with 650,000 inhabitants. These two outpatient services are organized on a catchment-area basis, and provide mental health care to the residents of the central region of the city. One of the services mainly provides medical-psychiatric monitoring in the form of regular consultations for people with different psychiatric diagnoses and more stable symptoms. The other offers more intensive multidisciplinary care for people with diagnoses of severe mental disorders and weaker social support networks, providing various activities throughout the day. 

A clinical convenience sample was composed without the participation of the service professionals in this selection. The criteria for inclusion in the study were: to be attending the selected services, in the period of data collection; to be over 18 years of age; and to be able to provide informed consent and reliable information. The exclusion criteria were: to present clinical features of organic brain disease or acute psychotic symptoms. A total of 308 psychiatric outpatients were approached, 57 (18.5%) refused to participate, citing that they had other commitments at the time and therefore did not have the available time to respond to the instruments, while 08 (2.6%) fulfilled the exclusion criteria. The sample was composed of 243 patients. After the data collection, all participants received brief interventions.

The instruments were selected with the aim of covering different psychosocial and emotional characteristics of the participants that could be associated with the problematic use of drugs: 

*Sociodemographic and clinical information questionnaire*: a standardized questionnaire was used to gather sociodemographic, social network, social harm, and clinical information. Variables, such as gender, age, marital status, religious practice, education, employment, personal income, financial satisfaction, length of psychiatric treatment, use of psychiatric medicines, problems with medicine use, history of psychiatric hospitalizations, drug use in the family, satisfaction with the community of residence, experiences of discrimination, history of violence, experience of living on the street and problems with the police, were investigated. The primary psychiatric diagnosis was retrieved from the medical records of the patients. 

*Alcohol, Smoking and Substance Involvement Screening Test (WHO-ASSIST)*: the WHO-ASSIST was chosen due to it being quick, reliable, valid and recommended for use with psychiatric patients. It also allows the evaluation of the problematic consumption of different substances, providing a more accurate picture of problematic drug use among individuals undergoing psychiatric treatment in community mental health services. The ASSIST instrument was validated in Brazil^8^. From the sum of the scores of the items related to the consumption of each substance, the individual is classified as a low-risk, moderate-risk or high-risk user, for the evaluated substance. In this study, a variable named Problematic Drug Use (PDU) was created, with all the patients that were classified as moderate risk or high-risk users for any of the drugs being classified as 1, and all the patients that did not make use of any of the drugs screened for or presented low-risk consumption with 0. This variable was considered as the dependent variable and created taking into account an international study that used ASSIST to investigate drug use among psychotic patients^9^. 

*Barratt Impulsiveness Scale (BIS-11)*: instrument used for the evaluation of Impulsivity, adapted for use with adults in Brazil^10^. The BIS-11 is a self-report scale consisting of 30 items related to the manifestations of impulsivity. The score of the scale items varies from 1 to 4, ranging from rarely/never to almost always/always and the score total of the instrument varies between 30 and 120. According to suggestions of the literature the total score of 72 or above was used to classify an individual as highly impulsive^11^. 

*Holmes and Rahe Stress Scale*: used to evaluate events in the previous year. This instrument was chosen because it has been adapted in Brazil for use with psychiatric patients. It contains 26 items^12^. The scale is based on the hypothesis that the effort required for the individual to readjust to society, after significant changes in life, creates deterioration that can lead to illness. The Social Readjustment Scale measures six categories of life events: work, loss of social support, family, environmental changes, personal difficulties, and finances. 

The data were collected by research assistants, who were graduate and undergraduate psychology students trained in clinical research methods, with weekly supervision by the coordinator of the project. They attended the mental health services on different days of the week and at varying times, inviting patients awaiting care to participate in the study. All the instruments were administered by the research assistants, with duration of 30 to 45 minutes per patient. The research assistant explained the research and the patients that agreed to participate were taken to a room within the service, where the instruments were applied.

 The research project was presented to the teams of the two services, who authorized the performance of the study and provided the physical space for the application of the instruments. The study received ethical approval from the Research Ethics Committee of the Faculty of Philosophy, Sciences and Letters of Ribeirão Preto of the University of São Paulo and all the participants signed the informed consent form. This report followed the STROBE Statement, using the checklist of items that should be included in reports of observational studies.

The data were double entered and organized, and both descriptive and inferential analyses were performed using the Statistical Package for the Social Sciences (SPSS) version 16 (SPSS, Chicago, IL, USA). The hypothesis of normality was verified for the numeric variables with categories with N < 50 (Shapiro-Wilk test). When normality was not rejected, the independent-samples t-test was used. For the categorical variables, the chi-square test was used to test for associations. To measure the strength of association between the categorical variables and PUD, the crude odds ratios were calculated considering a confidence interval of 95%. Next, in order to establish the adjusted odds ratios, stepwise multiple logistic regression was performed, aiming to determine the more important PUD predictors among the sample population, with a p-value <0.05 being considered significant.

## Results

The primary diagnoses of the patients that composed the sample, according to the International Classification of Diseases (10th version), were: organic mental disorders (n = 6; 2.5%), schizophrenia (n = 46; 18.9%), bipolar disorder (n = 26; 10.7%), depression (n = 76; 31.3% ), anxiety disorders (n=46; 18.9%), personality disorders (n = 3; 12.8%) and mental retardation (n = 12; 4.9%). The most frequent problematically used drug was tobacco, followed by alcohol (Table 01). Among the participants, 112 (46.1%) presented problematic use of at least one drug evaluated by the ASSIST. 

**Table 1:** Distribution of the problematic drug use by type of substance used. Ribeirão Preto, SP, Brazil, 2014

Substance	n	%
Tobacco	90	37
Alcohol	33	13.6
Cannabis	09	3.7
Cocaine	05	2.1
Amphetamines	02	0.8
Inhalants	04	1.6
Hallucinogens	02	0.8

The majority of the participants were women (71.2%), with ages ranging between 21 and 83 years, with a mean age of 48.2 years (SD = 13.2). The t-test for independent samples was used to compare the mean ages of the groups of patients who presented or did not present problematic drug use. No statistically significant difference was found (*t* = 1.65; *p* = 0.10). Considering marital status, 143 (58.9%) did not have a partner, although 196 (80.7%) reported that they lived with their family. Among the participants, 140 (57.6%) had only fundamental education or less. The majority did not work at the time of the study, being unemployed, retired or housewives (*n* = 159; 65.4%). Statistically significant associations were observed between problematic drug use and marital status, with this being more frequent in those who did not live with a steady partner. Statistically significant associations were also observed between problematic drug use and religious practice, in which this use was more frequent in those who did not practice any religion, and between problematic drug use and dissatisfaction with the financial situation (Table 2).

**Table 2:**
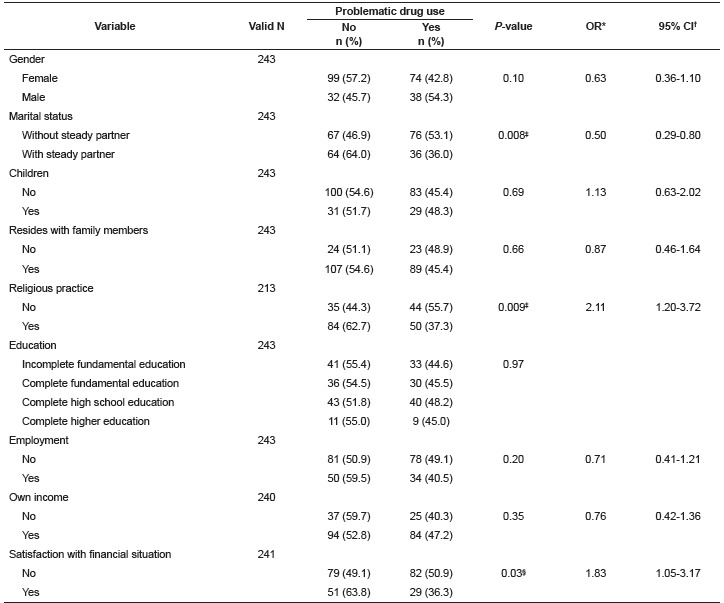
Sociodemographic characteristics of the psychiatric patients according to the presence or absence of problematic drug use. Ribeirão Preto, SP, Brazil, 2014

* Odds Ratio; † Confidence Interval; ‡ p < 0.01; § p < 0.05

The participation of the patient in different activities that could expand their social network were investigated, such as sports activities, participation in unions, political parties and NGOs, voluntary work and self-help groups. Whether the patients considered that they had someone to count on in moments of need, as well as their satisfaction with the community in which they resided were also investigated. Statistically significant associations were observed between problematic drug use and the participation in sports activities in the previous twelve months. Statistically significant associations between problematic drug use and dissatisfaction with the community in which the patient resided were also observed (Table 3).

**Table 3:**
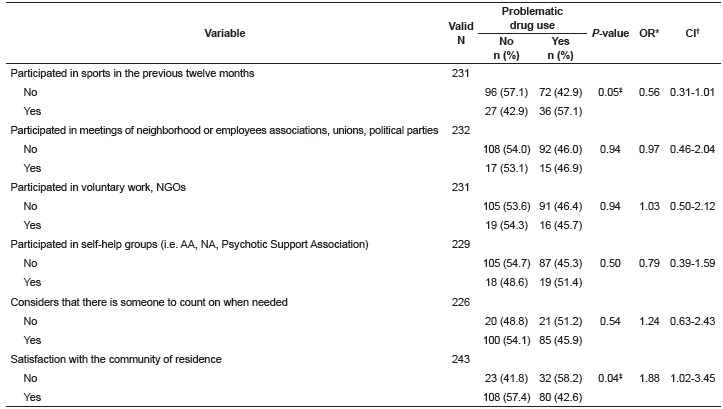
Social network of the psychiatric patients according to the presence or absence of problematic drug use. Ribeirão Preto, SP, Brazil, 2014

* Odds Ratio; † Confidence Interval; ‡ p ≤ 0.05

It was sought to evaluate whether problematic drug use was associated with some clinical features, such as a history of problematic drug use among family members, use of psychiatric medications and difficulties in adherence to this treatment, a history of psychiatric hospitalizations, duration of psychiatric treatment, stress events in the previous year, and impulsivity. The association of problematic drug use with social impairment, such as a history of violence, discrimination, living on the streets, and problems with the police, was also evaluated. Through the t-test significantly higher values were found for the mean duration of treatment (*t* = 2.59; *p* = 0.01) and the mean number of stress events in the previous year (*t* = 3.00; *p* = 0.003) in the group that presented problematic drug use. There was also an association between problematic drug use and the variables related to having experienced discrimination, the experience of living on the streets and a history of problems with the police (Table 4).

**Table 4:**
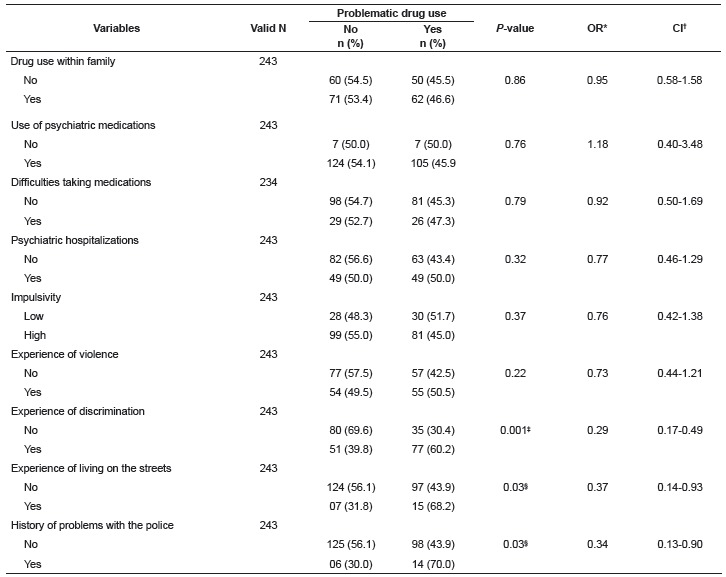
Clinical characterization and social impairments of the psychiatric patients according to the presence or absence of problematic drug use. Ribeirão Preto, SP, Brazil, 2014

* Odds Ratio; † Confidence Interval; ‡ p < 0.001; § p < 0.05

The variables listed in the previous tables, that obtained *p* < 0.10 were placed in a multiple logistic regression model (gender, age, religious practice, marital status, satisfaction with financial situation, participation in sports activities, satisfaction with the community of residence, duration of treatment, stress events in the previous year, experience of having suffered discrimination, lived on the streets, history of trouble with the police). The variable "previous psychiatric hospitalizations" was also added, as this is cited in the literature as associated with problematic drug use among psychiatric patients^2^. 

The variables that were found to be predictors of problematic drug use in the sample were marital status, religious practice, satisfaction with financial situation, experience of having suffered discrimination, and participation in sports activities in the previous year. Thus, according to this model, not living with a steady partner, not practicing religion, dissatisfaction with the financial situation, have suffered discrimination and having participated in sports activities are risk factors for problematic drug use among psychiatric outpatients (Table 5). 

**Table 5:** Variables that remained after multiple logistic regression. Ribeirão Preto, SP, Brazil, 2014

Variables	*P*-value	OR*	CI^†^
Marital status	0.028^‡^	0.491	0.260-0.927
Religious practice	0.016^‡^	0.449	0.234-0.862
Satisfaction with financial situation	0.047^‡^	0.469	0.249-0.990
Suffered discrimination	0.001^§^	3.821	2.02-7.23
Participated in sports activities in previous 12 months	0.022^‡^	2.25	1.12-4.50

* Odds Ratio; † Confidence Interval; ‡ p < 0.05; § p <0.001

## Discussion

 This study aimed to examine associations between sociodemographic, clinical and contextual characteristics and problematic drug use among psychiatric outpatients of community mental health services. Even with the division in the care network between mental health services and services specializing in the care of problematic drug users, most mental health services attend people that consume substances^6^. Knowing the characteristics of these people can help in establishing prevention and intervention strategies. In this study, characteristics related to the social context of the patient, such as marital status, religious practice, practicing sports, dissatisfaction with the financial situation, and having suffered discrimination were those shown to be risk factors for problematic drug use. 

It was found that not residing with a steady partner, whether married or cohabiting, was a risk factor for problematic drug use in this population. This information confirms the findings of other studies in the drugs area, with marital status being associated with a higher relapse rate among people with psychiatric diagnoses^13^. A Canadian population survey also highlighted that people with a partner were more likely to have positive mental health than those who were widowed, separated, divorced or single^14^. However, the results differ from those found in a study of psychiatric patients in Tanzania, where the authors observed there were no differences in alcohol use according to marital status^5^. To reside with a steady partner appears to protect individuals from problematic drug use, since the partner can assist in coping with stressful situations, without the subject having to resort to drugs. Furthermore, in Brazil, many psychiatric patients have their autonomy controlled by family members, who take care of the money and with this, control the use of substances^15^. A lack of religious practice has also been shown as a risk factor for problematic drug use. It is noteworthy that religion provides beliefs and explanations that assist in coping with situations of stress, which is associated with positive mental health^14^. Furthermore, when individuals practice a religion, they are usually part of a large social support network, which helps in everyday situations and also exerts behavioral control. 

It was observed in this study that only 34.6% of the participants were working at the time of the interview, despite the mean age of 48.2 years, i.e., a working age sample. Also noteworthy is that 25.8% of the respondents reported having no income of their own. Accordingly, it was hypothesized that many of the people could have been receiving welfare benefit or had been forced to retire early due to the psychiatric disorder. However, 66.9% of the respondents reported dissatisfaction with their financial situation, with this variable being identified as a risk factor in the multiple regression model. It is important to evaluate the social conditions of the patients in the mental health services and to propose actions that go beyond the reduction to the symptoms, i.e. the nursing team and other professionals of the services need to work in an intersectoral way, together with the social care and income generation services^16^. 

In this study it was found that practicing sports activities was another risk factor for problematic drug use in this population. At first this may seem strange, as authors highlight that practicing sports can serve as a protective factor with regard to drug consumption^17^. However, the literature is also controversial, and it has been found that, among adolescents, practicing sports is associated with the abusive use of alcohol^18^. It is considered that cultural aspects should be taken into consideration for comprehending the relationship between practicing sports and using drugs. In Brazil, the most popular sport is football. This is strongly associated with the consumption of alcoholic beverages, with it being common for friends to meet weekly to play football and drink beer after the game. It is common that this consumption is seen as a socialization resource, encouraged by the media and performed in an abusive way. Thus, the data found in this study deserve closer inspection, i.e. new investigations that seek to explore in greater detail the association between practicing sports and using drugs in this population. 

To have experienced discrimination was also indicated as a risk factor for problematic drug use among psychiatric patients in this study. Daily discrimination alone, for example, due to the skin color of the individual, can lead to increased depressive symptoms, showing a relationship between discrimination and mental health^19^. In addition, to feel discriminated against or stigmatized is very common among people with psychiatric diagnoses. The literature explains that part of this is due to the difficulties that the individual has in coping with their obligations, such as work. It is common for people with mental disorders to have higher rates of absenteeism, to make greater use of prolonged sick leave and to retire earlier^20^. This situation can generate more stress in the life of the individual, who is already vulnerable due to psychopathological symptoms, increasing the risk of seeking relieve from these emotions through drug use. It is suggested that studies be conducted to better understand the influence of discrimination on problematic drug use among psychiatric outpatients. This study indicates the importance of the mental health services addressing this subject among users, in order to help them deal with this issue and prevent worsening of the prognosis. 

One of the limitations of this study refers to the information on drug use being obtained through patient response, with medical records or other informants not consulted and biological tests not carried out to verify the accuracy of such information. Care was taken to provide the subjects with privacy and comfort, putting them in a private room and using the self-application technique. However, this is a sensitive subject to approach and there may have been people who chose not to reveal their substance consumption. Also, due to being self-applied, there were losses where some questions were not answered by some patients. Another limitation is the option to work with a non-randomized sample. Although this type of sampling is recommended for verifying whether a problem exists in a given place and is considered a way to obtain useful preliminary information concerning a particular health issue, these data cannot be used to estimate the prevalence of the event in the general population^21^. In this study, following international research recommendations^7^, we chose to work with a variable that encompasses the problematic use of any of the substances evaluated in order to provide preliminary data in Brazil regarding the problem of drug use among people undergoing treatment in community mental health services. In order to deepen the comprehension of the subject, further studies are recommended to investigate the consumption of each of the substances among individuals in outpatient psychiatric treatment. 

## Conclusions

This study aimed to cover a gap in the Brazilian literature related to the study of problematic drug use among people undergoing treatment in community mental health services. In this study, socio-demographic, clinical and social setting variables were evaluated and those found to be predictors were related to the social context of the patient. Risk factors, such as marital status, religious practice and practicing sports, draw attention to how aspects related to the social support network and the need for socialization play an important role in the use of drugs among psychiatric patients. Financial dissatisfaction and experiences of discrimination also stood out as predictors for the use of drugs. Thus, it is recommended that mental health services valorize psychosocial actions, seeking to know the social support network of patients, their modes of socialization, their financial needs, and their experiences of life and suffering. With this, multidisciplinary and intersectoral actions can be planned that aim to overcome the reduction to psychopathological symptoms. Considering nursing in particular, the results indicate the need for nurses to be aware of the characteristics of the context of the patient, in order to identify and plan actions directed toward problematic drug use, aiming to providing integral care for the user and establish priorities in the care plan. Furthermore, the importance of public policies that address the perception of the general population regarding mental disorders was highlighted, seeking to construct communities that are more tolerant to differences and to better comprehend individual vulnerabilities.
